# Attention Skills in Children With Nonsyndromic Cleft Lip and Palate

**DOI:** 10.1177/10556656251351756

**Published:** 2025-08-21

**Authors:** Stephanie van Eeden, Cristina McKean, Helen Stringer

**Affiliations:** 1 School of Education, Communication and Language Sciences, 5994 Newcastle University, Newcastle upon Tyne, UK; 2 Department of Education, University of Oxford, Oxford, UK

**Keywords:** cleft lip and palate, auditory attention, visual attention, speech, language

## Abstract

**Objective:**

To describe attention skills in children with cleft lip and palate across auditory and visual domains and investigate associations with cleft subtype, biological sex, socioeconomic status, and speech and language.

**Design:**

A cross-sectional observation study. Setting Participants from regional cleft lip and palate centers seen in schools and home.

**Participants:**

Eighty-one children aged 5 years 0 months to 7 years 11 months with non-syndromic cleft palate +/- lip. Main outcome measure Auditory and visual attention skills including measures of distractibility, impulsivity and focus across both domains.

**Results:**

Mean average scores for all measures of auditory and visual attention were below one standard deviation from the normative average (standard score <85). There was no main effect of cleft subtype. Boys had significantly lower scores for visual distractibility than girls. There was no statistically significant effect of socioeconomic status, but those from higher socioeconomic groups had scores up to 10 standard points higher in some measures than those in the middle or lower groups. Weak, significant correlations were observed between language skills and measures of attention.

**Conclusions:**

A range of both auditory and visual attention skills were low in this group raising the question of potential generalised attention difficulties in children with CLP. Attention skills were correlated with language. Professionals working with children with CLP should be aware that attention difficulties are prevalent and advise families and educators accordingly. Further research including intervention studies would increase our understanding.

## Introduction

Children born with cleft lip and palate (CLP) are known to be at risk of speech and language difficulties as well as lower educational achievement compared to their peers.^1,2^ Attention deficits have been linked with poor outcomes in speech, language, and education.^3–5^ There has been a global increase in interest in attention deficit disorders in the general population over the last 30 years. Rates of diagnosis for Attention Deficit Hyperactivity Disorder (ADHD) vary by country but there is a trend for increased prevalence.^6^ For example, a national population sample in the United States found an increase in prevalence in children from 6.1% to 10.2% between 1997 and 2016^7^ while in the United Kingdom, prevalence is reported to be lower but with a 2- to 3-fold increase in children between 2000 and 2018.^8^ There have been a number of studies looking at rates of ADHD in the CLP population, as well as some observational studies reporting sustained attention. However, findings have been contradictory with little detailed observation of different types of attention skills and how these relate to speech and language development.

Attention skills involve cognitive processes that allow us to focus on specific stimuli or tasks, maintain concentration, and manage multiple tasks. These skills are crucial for learning, working, and navigating daily life. Development begins in infancy but continues into adolescence.^9^ Attention skills are varied and include the ability to focus on a task without getting distracted, sustain attention, shift attention, and control impulses. Attention Deficit Hyperactivity Disorder is a neurodevelopmental condition that affects all these attention skills to a clinical level and can impact learning, social interactions, and well-being.^10,11^ Not all people with attention difficulties will be diagnosed with ADHD.

Studies of attention skills in people born with CLP have included population database studies investigating the risk of ADHD in this population, parental reports, and observational studies of sustained attention, thereby providing a range of evidence for both diagnosed ADHD and observed problems with attention. A Scandinavian study estimated a prevalence of ADHD in children with CLP of 8.9%^12^; this compares to an estimation of under 5% in the general population of Europe.^6^ A Taiwanese study compared 1158 children and adolescents with CLP with 11 580 noncleft peers and reported the likelihood of a diagnosis of ADHD over 7 times greater in the CLP group, with an average age of diagnosis being 8.6 years (HR: 7.33, 95% CI: 5.01-10.73).^13^ In a similar study, with children under the age of 5 years, a Swedish study reported only a slight increase in ADHD diagnosis compared to noncleft peers (aHR: 1.25, 95% CI:1.08-1.46).^14^

Reported rates of ADHD in people with CLP have been as high as 24%,^15^ although this has been disputed by some researchers. Richman et al^16^ investigated the diagnosis of ADHD in 177 children with CLP aged 7 to 12 years old. Their aim was to investigate this diagnosis alongside consideration of language skills. They reported that 18% (n = 32) of the cohort had been given a diagnosis and were receiving medication for ADHD prior to their investigation. They carried out language and cognitive assessments to reconsider diagnosis, after which they reported that only 10 of the 32 who were originally diagnosed exhibited clear features of ADHD; 21 had a language-based learning disability which accounted for attention difficulties. Only 5 of these reached thresholds for ADHD as well and at follow-up 6 to 9 months later, all 5 of these children had received language support and had discontinued medication for ADHD. Final estimates of the prevalence of ADHD were 6%, in line with the general population in North America at that time.^6^ This study highlights the difficulties when discussing attention skills and the need to be clear about the clinical features of ADHD and other observed difficulties with attention.

Studies using parent questionnaires often report very high rates of hyperactivity and attention problems in children with CLP. A recent study examining parent questionnaires in a UK cohort found that children with CLP aged 5 to 7 years were more than twice as likely to exhibit hyperactivity compared to the general population.^17^ Ma et al's study^18^ of 147 children aged 6 to 15 years found 40.7% of parents of children with isolated cleft palate reported attention difficulties compared with 27.7% of those with CLP and only 10% of those in an age-matched noncleft control group. This shows a potential effect of cleft subtype.

Smaller observational studies looking at sustained attention have added to our current understanding of attention skills without a diagnosis of ADHD. Lemos and Feniman^19^ studied 55 children with CLP aged 7 to 8 years, with no diagnosis of ADHD. In a measure of sustained attention, the participants were presented with 6 sets of 100 words and asked to indicate when they heard the target word. The researchers reported no differences in focus or impulsivity between participants with CLP and aged-matched noncleft peers but observed a significantly lower score for sustained attention over the course of the assessment. Maximino et al^20^ also reported that 11 of 22 children aged 7 to 9 years with unilateral CLP (UCLP) failed the same measure of sustained attention. In this study, up to 50% were also reported to have concomitant language difficulties. These observational studies all consider auditory attention skills. Children born with CLP are at high risk of conductive hearing loss in the early years.^21^ It could therefore be hypothesized that it is likely to be the auditory domain in which we might observe attention deficits in this population.

Visual attention skills, however, also influence developmental trajectories including language development.^22^ The role of joint attention between infant and parent has long been proposed as a necessary component for language learning,^23,24^ and the importance of visual skills in the development of early categorization of objects as the building blocks to labeling and development of vocabulary has more recently been purported.^25^ Understanding how attention can influence speech and language skills should therefore include both study of auditory and visual skills. To the best of our knowledge, there is no existing literature that addresses visual attention skills in children with CLP.

Studies of attention skills in children with CLP to date have provided contradictory findings depending on the study design, outcome measures, and consideration of comorbid diagnoses. There may be some aspects of attention skills which do not necessarily fall within the clinical boundaries of a diagnosis of ADHD, but which may be important to consider when speech and language development is a key outcome of interest as in children born with CLP. Further research into specific attention skills in this population is needed, including exploration of different stimuli and the relation of attention to speech and language skills. The study reported here aimed to explore and describe distractibility, impulsivity and focus across the 2 domains of auditory and visual attention.

## Aim

To explore auditory and visual attention skills in children aged 5 to 8 years with CLP.

### Research Questions

1. Do children aged 5 to 8 years with CLP have age-appropriate auditory attention skills?
2. Do children aged 5 to 8 years with CLP have age-appropriate visual attention skills?
3. Are there any observable differences between auditory and visual attention in children aged 5 to 8 years with CLP?
4. Are there any patterns of deficit in terms of distractibility, impulsivity, and focus across both domains?
5. Are there any group differences between different cleft subtypes, males and females, and low and high socioeconomic status?
6. Is there a correlation between attention skills and speech and language?

## Ethics

Full ethical approval was granted through the NHS Research Ethics Committee (19/ES/0071) and the Health Research Authority in the United Kingdom.

## Method

### Design

This was a cross-sectional observational study.

### Participants

Participants were recruited to a larger study exploring language and auditory skills in children born with CLP (Cleft Language and Auditory Skills [CLAS] study).^26^ Data on attention skills were collected as part of this study and are reported here. Participants were recruited from regional CLP teams across the United Kingdom. There were 95 participants in total. The inclusion criteria were children aged 5 years 0 months to 7 years 11 months with cleft palate only (CP) (including those with Robin Sequence), UCLP, and bilateral CLP (BCLP). All participants were nonsyndromic. Those with sensorineural hearing loss, known learning disabilities, and English as a second language were excluded.

### Procedures

The Test of Variables of Attention (T.O.V.A) v9^27^ was administered to assess attention to both auditory and visual stimuli. Stimuli were presented via a computer and the participants used a microswitch to record their responses. A Dell Vostro 15 3000 series laptop and over-ear headphones (Sennheiser Momentum 2) were used at a volume of 50% on the laptop for the auditory test. Tests were carried out in a quiet room on a one-to-one basis at school or home. This is as recommended in the T.O.V.A manual.

For the auditory test, participants listened to 2 tones presented randomly at different frequencies. They were asked to press a microswitch when they heard the high-frequency tone and ignore it when they heard the low-frequency tone. Using tones allowed assessment of attention to auditory stimuli without using words and introducing an element of linguistic processing. For the visual test, participants were required to watch the screen where visual stimuli were presented in different locations. The participants were asked to press the microswitch when they saw the visual stimulus in the target location on the screen.

Both tests were preceded by a practice test to ensure the participants understood the procedure. This included a description of what the tasks entailed as outlined in the manual followed by a trial task lasting 2 min. If participants appeared to struggle, they were able to repeat the trial task once more. Only when it was apparent that the participants had understood what both the visual and auditory targets were, either by completing the trial task accurately or repeating back the instructions to the researcher, did the full trial go ahead. This led to some exclusions as outlined below. Timings were accurately measured to the millisecond. The T.O.V.A v9.0 uses hardware in a device which connects to the computer, bypassing computer operating systems thus allowing highly accurate measures of timing.^28^

The primary aim of the T.O.V.A assessment is to test for ADHD. The full assessment is 21 min for the auditory domain and a further 21 min for the visual domain. The participants in this study were also undertaking a large battery of language and auditory assessments. As we were not using this assessment for diagnostic purposes, only the first quarter of each assessment was completed (5.25 min for each domain; 10.5 min in total). This allowed for collection of a standard score measurement for stimuli that were presented infrequently as opposed to frequently. Infrequent presentation is more challenging than frequent presentation and according to the T.O.V.A manual is traditionally used for measuring vigilance.^27^ Each quarter has separate normative scores with which to compare outcomes. While sessions started with the speech assessment and 2 of the language subtests, tasks requiring attention skills were always carried out following a break of at least 10 min during which the children would go out to play, have a snack or play a game on the iPad. The limitations of this methodology are discussed further below.

### Outcome Measures

The primary outcome measures produced by the T.O.V.A and used in this study reflect different aspects of attention control. These are levels of distractibility (as measured by variability in response times), impulsivity (measured by number of commission errors, ie, pressing the microswitch for a nontarget sound or image) and focus (measured by number of omission errors, ie, failing to press the microswitch when the target sound or image was presented). Standard scores are given for each quarter of the assessment completed.

Other variables were included to explore the data further. These included cleft subtype, biological sex, socioeconomic status (derived from Index of Multiple Deprivation^29^ deciles and categorized into low, medium, and high), and speech (measured using Percent Consonants Correct [PCC] calculated from the Diagnostic Evaluation of Articulation and Phonology [DEAP]^30^ and language skills [measured using the Core Language Scale of the Clinical Evaluation of Language Fundamentals – 5^th^ Edition CELF-5^UK^], which provides a composite standard score reflecting both receptive and expressive language skills from the completion of subtests).^31^ Hearing was considered a potential confounder to results for auditory attention skills. Hearing levels were assessed on the day using the SHOEBOX^®^ QuickTest (v.5.6.5) audiometry screen. The SHOEBOX^®^ is a clinically validated hearing screen designed to be used outside of an audiometric booth.^32,33^ This screen tests, hearing across a range of decibel levels from 10 dB up to a top level of 55 dB for frequencies of 500, 1000, 2000, and 4000 Hz, provide a bilateral audiogram. The SHOEBOX^®^ determines that the screen is failed if a loss at any frequency over 25 dB is detected. Where participants were uncooperative with the assessment or there was equipment failure, data were obtained from an audiology report within 3 months of the assessment date. Therefore, a dichotomous pass/fail variable was used as a confounder in analysis.

### Data Analysis

SPSS v29^34^ was used for all statistical analyses. Normative data from the standardized test were used to compare data from this study to a normative sample rather than including a control group. The mean and median standard scores are reported along with standard deviations (SDs) and the interquartile range for the whole group and across cleft subtypes, biological sex, and socioeconomic status for all primary outcome measures. Floor effects were observed for some subtests as many participants scored at the lowest standard score of 40 on the T.O.V.A subtests which provided negatively skewed data. For consistency, nonparametric statistical tests were therefore used to explore group differences. Spearman correlations explored associations between all measures of attention and speech and language. Partial correlations were also carried out. These accounted for age when correlating with speech data as the PCC measure is not standardized and for hearing as a potential confounder to auditory outcomes.

Statistically significant differences or associations were determined at the *P* < .05 level in keeping with common convention. However, results were also interpreted according to more recent calls for a move away from dichotomizing results analyzing the strength of an association by considering *P*-values along with a continuum.^35,36^ Effect sizes are reported using Cohen *d* and r (small effect = 0.1; medium effect = 0.3; large effect = 0.5).^37^

## Results

### Sample

Out of the 95 participants recruited to the full CLAS study, 81 (85%) completed the attention assessments. Of the 14 participants who were excluded from the attention analysis, 4 either refused to do the assessment or said they were unable to cope with the visual stimuli. No data on diagnosis of or suspected ADHD were recorded as this was not the focus of the main study. Further exclusions were due to difficulties being able to distinguish between different sound frequencies to carry out the auditory assessment (n = 7) and equipment failure or environmental reasons for the test not being carried out (n = 3). Forty-four participants had a diagnosis of CP, 13 of whom also had a diagnosis of cleft palate with Robin Sequence (CPRS); 28 participants had a diagnosis of UCLP and 9 BCLP. Seventy-two participants (89%) had available data from audiograms taken either on the day or within 3 months of the assessment as detailed in “Methods” section. Of these, 42 (58%) had hearing within the normal thresholds. The ratio of male (n = 40) to female (n = 41) participants was almost 1:1. Participants were from different socioeconomic backgrounds with 39 categorized as low SES (1-3 deciles), 21 mid SES (4-7 deciles), and 21 high SES (8-10 deciles). The mean average age of participants was 6 years 6 months.

### Attention Skills

Full results from the descriptive statistics can be found in Table 1.

**Table 1.** Descriptive Statistics for All Measures of Attention Across the Whole Group and by Cleft Subtype.

	Cleft subtype	Number of participants	Distractibility^a^		Impulsivity^b^		Focus^c^	
			M (SD, min-max)	Mdn (IQR)	M (SD, min-max)	Mdn (IQR)	M (SD, min-max)	Mdn (IQR)
Auditory domain	All cleft subtypes	81	83.4 (24.3, 40-126)	90.00 (30.00)	84.3 (25.4, 40-122)	97.00 (30.00)	78.4 (25.0, 40-113)	82.00 (45.00)
	CP	31	77.6 (26.8, 40-121)	80.00 (61.00)	79.0 (27.6, 40-106)	96.00 (62.00)	73.8 (25.0, 40-112)	73.00 (54.00)
	CPRS	13	94.5 (12.1, 40-112)	93.00 (37.00)	98.2 (9.3, 73-108)	101.00 (7.00)	91.9 (22.9, 40-113)	100.00 (24.50)
	UCLP	28	88.0 (22.9, 40-126)	92.00 (25.75)	87.3 (23.4, 40-122)	96.50 (28.50)	80.0 (24.9, 40-109)	81.00 (45.25)
	BCLP	9	73.5 (27.0, 40-108)	84.00 (54.50)	73.4 (31.9, 40-104)	94.00 (63.00)	69.3 (23.4, 40-97)	80.00 (48.50)
Visual domain	All cleft subtypes	81	77.0 (25.6, 40-121)	79.00 (50.50)	85.0 (25.2, 40-114)	94.00 (29.50)	78.3 (24.3, 40-116)	84.00 (41.00)
	CP	31	72.7 (25.1, 40-114)	78.00 (53.00)	78.0 (27.7, 40-111)	89.00 (63.00)	71.8 (25.0, 40-109)	81.00 (54.00)
	CPRS	13	89.1 (22.4, 40-121)	83.00 (34.50)	96.0 (17.5, 51-114)	101.00 (19.00)	95.8 (10.3, 78-111)	96.00 (17.50)
	UCLP	28	80.0 (24.8, 40-119)	86.50 (35.25)	90.1 (21.2, 40-112)	95.50 (23.75)	81.8 (21.6, 40-116)	87.50 (25.75)
	BCLP	9	64.6 (26.8, 40-99)	46.00 (59.00)	77.4 (30.9, 40-109)	93.00 (69.00)	64.0 (30.2, 40-107)	40.00 (59.50)

a Variability in response time.

b Commission errors.

c Omission errors.

Abbreviations: BCLP, bilateral cleft lip and palate; CP, cleft palate only; CPRS, cleft palate only with Robin Sequence; UCLP, unilateral cleft lip and palate.

Mean standard scores for all measures of attention across both the auditory and visual domains were at or below one SD from the normative mean of 100 (SD 15) for the whole group. Ability to focus on both auditory and visual stimuli was below a standard score of 80 which represents a clinical cutoff point for this assessment.^28^ This was also the case for distractibility for visual stimuli. Median scores were higher than the mean in some instances reflecting the non-normal distribution of some of the data.

## Auditory Attention

All measures of auditory attention were at or below one SD from the normative mean of 100 (SD 15) for the whole group. The mean standard score for the ability to focus on auditory stimuli was 78.4 (SD 25), below a standard score of 80 which represents a clinical cutoff point for this assessment,^28^ but with a greater SD compared to the normative data.

Median scores were higher than the mean for distractibility (Mdn = 90.0; M = 83.4) and impulsivity (Mdn = 97.0; M = 84.3) measures. Floor effects on these subtests lowered the mean scores. The median standard score for the measure of focus on auditory stimuli was more similar to the mean (Mdn = 82.0; M = 78.4).

### Visual Attention

All measures of visual attention were at or below one SD from the normative mean of 100 (SD 15) for the whole group. The mean standard score for the ability to focus on visual stimuli was 78.3 (SD 24.3), below a standard score of 80 which represents a clinical cutoff point for this assessment,^28^ but with a greater SD compared to the normative data. The mean standard score for distractibility measures during tasks requiring attention to visual stimuli was also below the clinical cutoff point at 77.0 (SD 25.6).

As with the auditory subtests, some median scores were higher than the mean scores due to negatively skewed data. This was the case for visual impulsivity (Mdn = 94.0; M = 85.0) and visual focus to a lesser degree (Mdn = 84.0; M = 78.3). Median and mean scores for visual distractibility were both below 80 (Mdn = 79.0; M = 77.0).

Exploration of differences between auditory and visual attention was carried out using Spearman correlations. These showed high levels of correlation between skills: distractibility (r_s_ = .616, *P* < .001), impulsivity (r_s_ = .615, *P* < .001), and focus (r_s_ = .560, *P* < .001). A related-samples Wilcoxon signed-rank test showed that there were no differences observed between scores for the auditory and visual attention subtests for impulsivity and focus; however, scores for distractibility in the visual domain were significantly lower than in the auditory domain (see Table 2).

**Table 2.** Results of a Related-Samples Wilcoxon Signed-Rank Test Comparing Aspects of Attention Skill Across the Auditory and Visual Domains.

	z	*P*	Cohen’s *d*
Distractibility^a^	−2.492	.013	0.276
Impulsivity^b^	1.352	.177	0.150
Focus^c^	−.297	.766	0.033

a Variability in response time.

b Commission errors.

c Omission errors.

In exploring similarities and differences across the domains, we also sought to observe any patterns of deficit in terms of distractibility, impulsivity and focus across both domains. The ability to focus on both auditory and visual stimuli was impaired. The mean average score for ability to focus was below one SD from the normative mean standard score of 100 across both domains (M = 78.4 [auditory]; M = 78.3 [visual]). Signs of impulsivity were of less concern in both domains (M = 84.3 [auditory]; M = 85.0 [visual]). Mean standard scores for levels of impulsivity were just at the level of one SD below the mean but with higher median scores across both domains. Distractibility measures were lower for the visual domain as reported above.

## Exploring Group Differences

Group differences between cleft subtypes, males and females, and levels of socioeconomic status were analyzed using nonparametric statistical tests. The results from each of these groups are reported below.

### Cleft Subtype

**Figure 1.**
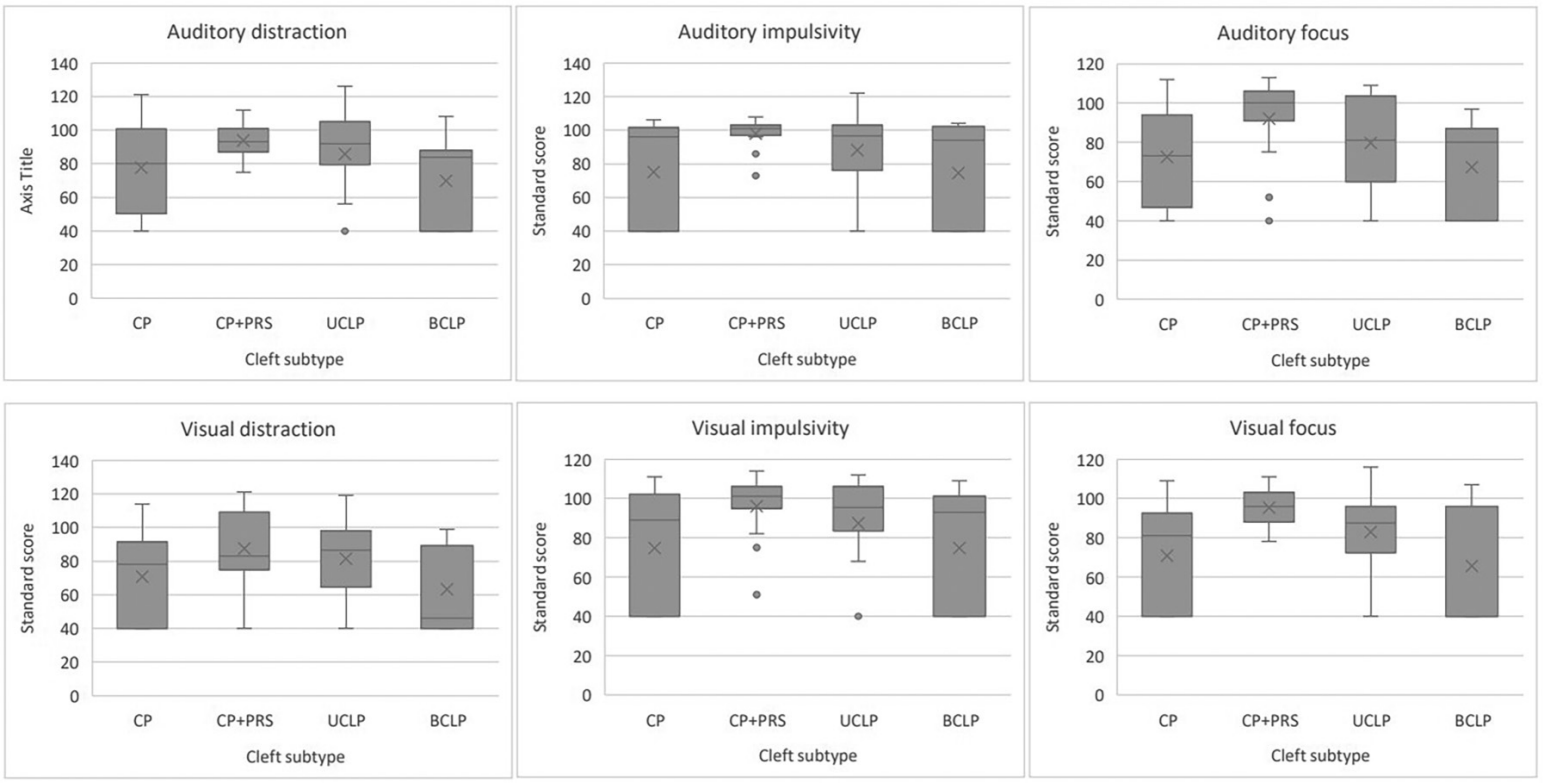
Box and whisker charts comparing mean (X) and median (-) standard scores across the cleft subtypes for all auditory and visual subtests. BCLP, bilateral cleft lip and palate; CP, cleft palate; CP+PRS, cleft palate and Pierre Robin Sequence; UCLP, unilateral cleft lip and palate.

Details of results by cleft subtype are shown in Table 1. Participants with CP or BCLP had the lowest scores; the mean standard score for both groups fell below a standard score of 80, the cutoff for clinical significance, for all measures. Median scores reflected this for distractibility and focus across both domains. Participants with a CPRS had scores within the average range expected for their age. This is further illustrated in Figure 1. A Kruskal-Wallis analysis with Bonferroni corrections showed statistically significant differences only between those with CPRS and either CP (H = −22.9, adj. *P* = .018) or BCLP (H = −26.8, adj. *P* = .049) in the visual focus measure only. The number of participants in the CPRS and BCLP groups was low, however. Combining cleft subtypes into 2 groups of CLP and CP showed no statistical group difference across any measures of attention.

### Biological Sex

The mean standard scores for both males and females were at or below one SD from the normative mean of 100 for all auditory and visual subtests. Median scores were higher than the means for impulsivity measures and auditory distractibility. Group differences were observed between the biological sexes in visual distractibility measures with males significantly more distractible than females (Mann-Whitney *U* test: *U* [1039.00; *P* = .037; r = .231]). Full results are shown in Table 3.

**Table 3.** Descriptive and inferential Statistics for All Measures of Attention by Biological Sex.

		Auditory domain			Visual domain		
		Distractibility^a^ M (SD, min-max); Mdn (IQR)	Impulsivity^b^ M (SD, min-max); Mdn (IQR)	Focus^c^ M (SD, min-max); Mdn (IQR)	Distractibility^a^ M (SD, min-max); Mdn (IQR)	Impulsivity^b^ M (SD, min-max); Mdn (IQR)	Focus^c^ M (SD, min-max); Mdn (IQR)
Sex (n)	Male (40)	83.2 (26.4, 40-126) 89.00 (42.75)	84.5 (26.5, 40-122) 97.00 (42.50)	77.4 (26.5, 40-112) 79.50 (57.25)	70.1 (26.0, 40-115) 72.00 (55.00)	85.7 (25.4, 40-112) 94.50 (31.75)	74.8 (25.9, 40-116) 83.00 (56.00)
	Female (41)	83.7 (22.5, 40-116) 91.00 (27.50)	84.1 (24.6, 40-108) 97.00 (68.00)	79.3 (23.8, 40-113) 83.00 (39.50)	83.7 (23.7, 40-121) 83.00 (30.00)	84.3 (25.3, 40-114) 93.00 (33.50)	81.6 (22.6, 40-111) 90.00 (71.00)
Mann-Whitney	*U*	802.500	761.500	831.000	1039.000	781.500	925.000
	*P*	.868	.578	.917	.037	.715	.318
	*r*	.489	.464	.506	.633	.476	.564

a Variability in response time.

b Commission errors.

c Omission errors.

### Socioeconomic Status

**Figure 2.**
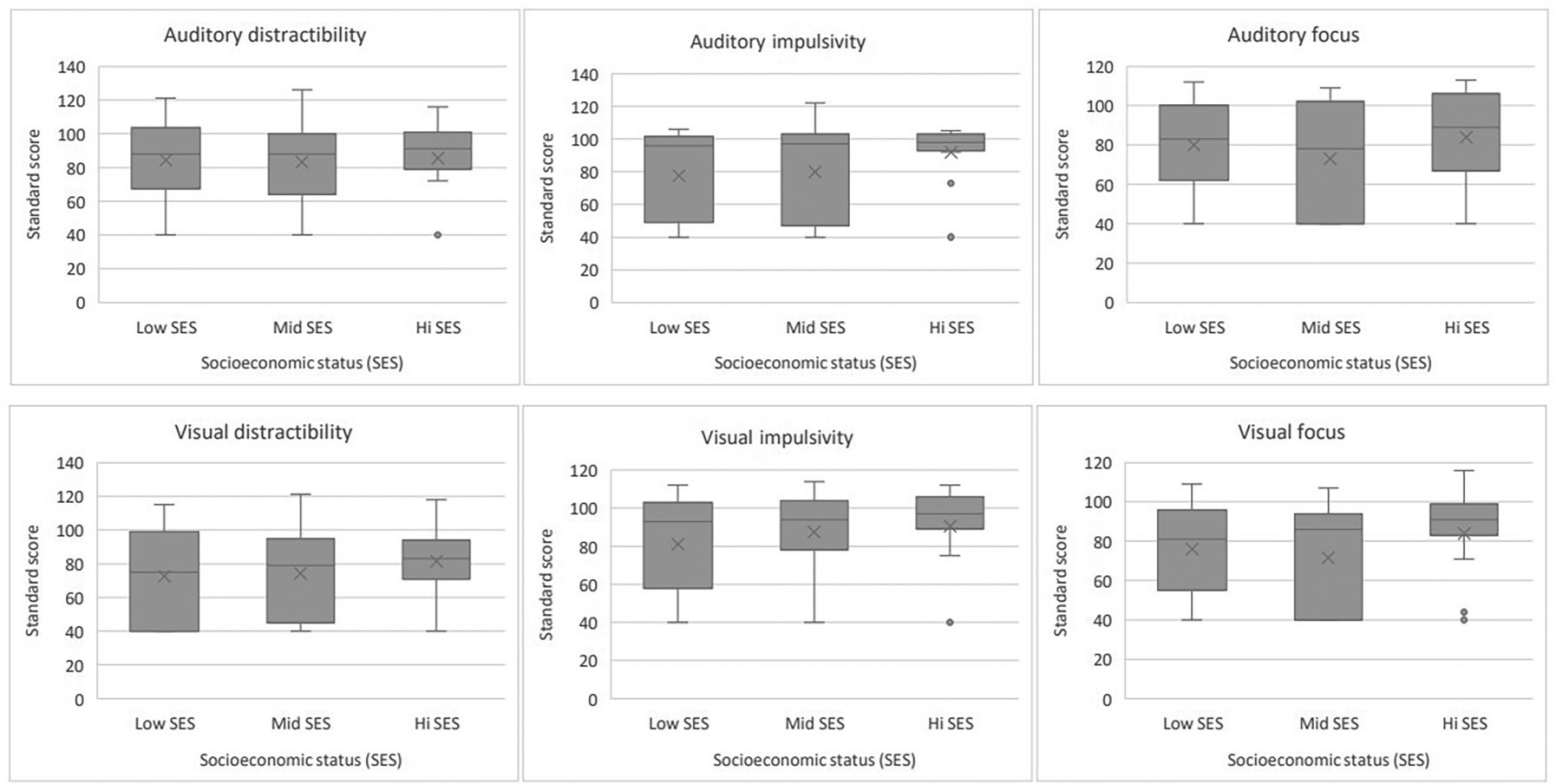
Box and whisker charts comparing mean and median standard scores across low, middle and high socioeconomic (SES) groups for all auditory and visual subtests. SES groups derived using *Index of Multiple Deprivation scores*^29^ - Low = deciles 1-3; Mid = deciles 4-7; High = deciles 8-10.

Higher scores were observed in the higher socioeconomic group (IMD deciles^29^ 8-10), with mean standard scores ranging 81.7 to 88.5 in the auditory domain compared to 78.7 to 82.5 in the lower socioeconomic group (IMD deciles 1-3) and 74.4 to 82.8 in the middle group (IMD deciles 4-7). In the visual domain, the higher socioeconomic group mean standard scores ranged from 80.7 to 92.5 compared with 74.8 to 81.7 in the lower group and 77.0 to 83.7 in the middle group. There were also some outliers in the high group. This is illustrated in Figure 2. No statistically significant differences were observed between the socioeconomic groups (see Table 4).

**Table 4.** Descriptive Statistics for All Measures of Attention Across Low, Mid, and High Socioeconomic Status.

	Socioeconomic status	Number of participants	Distractibility^a^ M (SD, min-max); Mdn (IQR)	Impulsivity^b^ M (SD, min-max); Mdn (IQR)	Focus^c^ M (SD, min-max); Mdn (IQR)
Auditory domain	Low^a^	39	82.5 (24.9, 40-121) 88.00 (43.00)	81.2 (26.3, 40-106) 96.00 (62.00)	78.7 (23.9, 40-112) 83.00 (39.00)
	Mid^b^	21	80.1 (27.3, 40-126) 88.00 (50.00)	82.8 (28.5, 40-122) 97.00 (59.50)	74.4 (27.2, 40-109) 78.00 (62.50)
	High^c^	21	88.5 (20.1, 40-116) 91.00 (24.00)	91.5 (19.2, 40-105) 98.00 (10.50)	81.7 (25.5, 40-113) 89.00 (47.00)
Kruskal-Wallis	H		0.896	1.402	1.016
	*P*		.639	.496	.602
Visual domain	Low^a^	39	74.8 (27.6, 40-115) 75.00 (63.00)	81.7 (26.8, 40-112) 93.00 (52.00)	75.7 (24.1, 40-109) 81.00 (41.00)
	Mid^b^	21	77.2 (26.9, 40-121) 79.00 (54.50)	83.7 (26.5, 40-114) 94.00 (45.00)	77.0 (25.5, 40-107) 86.00 (55.00)
	High^c^	21	80.7 (21.0, 40-118) 83.00 (25.00)	92.5 (19.8, 40-112) 97.00 (18.00)	84.3 (23.7, 40-116) 91.00 (24.00)
Kruskal-Wallis	H		0.400	1.680	2.241
	*P*		.819	.432	.326

^a^Low = deciles 1 to 3 of Index of Multiple Deprivation (IMD).^29^

^b^Mid = deciles 4 to 7 of IMD.

^c^High = deciles 8 to 10 of IMD.

## Relationship of Attention to Speech and Language

Spearman correlations were used to explore the relationship between attention skills and speech and language outcomes. These can be found in Table 5. Hearing was identified as a potential confounder to auditory attention skills. Partial correlations were conducted to account for hearing as a confounder to auditory results.

**Table 5.** Spearman and Partial Correlations Between All Measures of Attention, Speech, and Language Skills.

		Speech^d^			Language skills^e^		
		r_s_	*P*	Partial correlation controlling for age and hearing^f^	r_s_	*P*	Partial correlation controlling for hearing^f^
Auditory domain	Distractibility^a^	.163	.145	r_s_ = .162, *P* = .171	.149	.184	r_s_ = .252, *P* = .030
	Impulsivity^b^	.131	.242	r_s_ = .104, *P* = .379	.149	.184	r_s_ = .336, *P* = .003
	Focus^c^	.202	.071	r_s_ = .126, *P* = .287	.276	.013	r_s_ = .317, *P* = .006
Visual domain	Distractibility^a^	.174	.121	-	.275	.013	-
	Impulsivity^b^	.088	.435	-	.154	.171	-
	Focus^c^	.151	.178	-	.312	.005	-

a Variability in response time.

b Commission errors.

c Omission errors.

d Percent Consonant Correct (PCC) score calculated from Diagnostic Evaluation of Articulation and Phonology.^30^

e Core Language Scale from Clinical Evaluation of Language Fundamentals – 5^th^ Edition.^31^

f SHOEBOX^®^ QuickTest (v.5.6.5).

### Speech

Speech was measured using a PCC score from the DEAP (Dodd et al., 2002). There were no statistically significant correlations between speech and any measures of attention skills after controlling for hearing and age.

### Language

Language skills were measured using the core language scale from the CELF-5^UK^.^31^ Language was correlated with the ability to focus in both domains (auditory: r_s_ = .276, *P* = .013; visual: r_s_ = .312, *P* = .005) as well as distractibility in the visual domain (r_s_ = .275, *P* = .013). These correlations represented a weak but statistically significant relationship. A partial correlation controlling for hearing showed a statistically significant weak correlation between language and all auditory attention measures (see Table 5).

## Discussion

This study explored the attention skills of children with nonsyndromic CLP across both the auditory and visual domains. It described levels of distractibility, impulsivity, and focus. It investigated differences across cleft subtypes, biological sex, and socioeconomic status. It also looked at the relationship to speech and language.

Mean average levels of attention skill in this group were low across all measures in both the auditory and visual domains. A range of scores above and below the mean was observed reflecting different levels of skill for different participants. Standard deviations were larger than the normative sample reflecting more variability in this group of participants. For measures of focus, mean standard scores fell below one SD (<85) from the normative mean of 100 for both auditory and visual stimuli. This was also the case for measures of distractibility in the visual domain. Impulsivity was the only measure with a mean score near the range expected for this age group. Moreover, no clear differences were observed between auditory and visual attention, with all measures across both domains highly correlated. Only distractibility with visual stimuli showed a significant difference compared to the related auditory measure. This suggests that there was a generalized level of difficulty observed in this group and not a specific difficulty with auditory attention as may have been hypothesized given the high level of hearing impairment experienced by this population in early childhood.^21^

There was no main effect of group when results across the cleft subtypes were analyzed. There were small numbers in the CPRS and BCLP groups which means interpretation needs due caution. However, analysis of broader groups (ie, CP compared to CLP) also showed no significant group differences which is similar to findings in most other studies,^12,15^ with only one finding a higher incidence in children with CP compared with other cleft subtypes.^14^

Boys had significantly lower scores than girls for measures of visual distractibility. Scores for auditory and visual focus were also low for boys, although with no statistically significant differences between boys and girls observed. Boys have been reported to be diagnosed with attention difficulties more frequently than girls in the general population,^6,7^ but other studies comparing outcomes by biological sex in those with CLP have found no differences.^12,15^ Given that we only found one measure that was different we can conclude that biological sex is unlikely to have an effect on most attention skills.

We found no statistically significant effect of socioeconomic status. However, participants in the higher socioeconomic groups consistently scored better than those in low or mid socioeconomic groups, by up to 10 standard points in some incidences. In a general population study, Xu et al^7^ found significantly higher rates of diagnosis of ADHD in the lowest socioeconomic groups in the United States. Other observational studies have also shown links between socioeconomic status and the development of attention skills.^38^ However, in a population database study in Taiwan, no effect of socioeconomic status as measured by location of residence and income was observed.^13^ The relationship with socioeconomic status is likely to be more complex with known links with language skills, parental behaviors, and childhood trauma playing a part.^38–40^

This study explored links with speech and language skills through correlation of attention measures with PCC and a core language scale, combining both receptive and expressive skills. No statistically significant correlation was seen with attention skills and speech as measured by PCC. Few studies in the wider speech disorder literature have investigated links between speech difficulties and attention problems but there is some evidence to suggest a potential comorbidity,^3,41^ with children presenting with both speech *and* language difficulties more likely to have concomitant attention problems.^42^ A weak but significant correlation with the core language scale and auditory and visual focus was observed, as well as with visual distractibility. After controlling for hearing status in the auditory domain, evidence for a correlation between language and all auditory attention measures was observed. Richman et al reported a high level of language-based learning difficulties in their group of children aged 7 to 12 years who had previously been diagnosed with ADHD, showing how it can be difficult to disentangle attention difficulties from language skills.^16^ It is easy to comprehend how auditory attention and speech and language development relate, perhaps less so visual attention. However, studies have shown that infants learn early categorization skills through visual attention.^25^ This can be thought of as the early building blocks of the lexicon. Auditory skills, in terms of labeling the objects they see, aid this process^43,44^; but it is the initial visual attention skill that is key and distracting noises may even interfere with the infant's ability to attend and store visual information in order to build strong categories, as attention skills are diverted.^45^ While we found correlations, this does of course not tell us anything about causation. Further research in this area is needed.

The impact of hearing status on attention is another area of interest. Losses in peripheral hearing can affect the ability to filter out sounds and selectively attend to stimuli.^46^ Findings from studies into attention skills in children with peripheral hearing loss are mixed, with many investigating small numbers of participants with sensorineural hearing loss. For example, a small study of hearing impaired preschoolers found some difficulties in attention skills compared to hearing peers^47^; this has also been found in older children.^48^ However, a larger survey of older children found little increased risk of attention diffculties,^49^ and another study found no differences in levels of visual attention.^50^ This study did not focus on hearing and hearing status in this study was determined by a simple screen and so direct comparisons are not possible to others; furthermore, measures of attention vary widely in the literature. Hearing levels should be considered in future studies of attention in the CLP population.

## Strengths and Limitations

While there are several studies which have looked at ADHD in children with CLP which use checklists or have interrogated databases,^12–15^ there is limited study investigating the detail of attention skills of people born with CLP and specifically how these relate to other outcomes of interest in this population, namely speech and language. This study compared both auditory and visual skills so that we were able to describe specific abilities.

The cross-sectional design allowed exploration and description of data across a good overall sample size, but analysis of cleft subtypes meant that some groups were very small making it difficult to reach definite conclusions.

The data reported in this paper are from a larger study. This allowed exploration of not only specific attention skills but also their relationship to speech and language. This was also one of the limitations of this study. Participants in this study had to complete a large battery of assessments. This meant that the full T.O.V.A test was not carried out which means validity must be considered with caution; the T.O.V.A was also not carried out as the first assessment of the session in the morning as advised in the manual. This opens the possibility of fatigue affecting the results. The study design aimed to mitigate the effects of fatigue by ensuring the attention tasks were given after a break. We cannot be certain that if the participants had completed the attention tasks on a separate occasion they would have performed the same, but all participants had the assessments presented in the same order and we observed a range of scores around the mean reported here. It is possible that the floor effects observed here were due to an overload of assessment tasks. This meant that not all data were normally distributed and so we were unable to reliably use parametric statistical analysis to further interrogate and understand the relationships between the variables. Future research focusing solely on attention skills in a larger study will improve on this methodology.

As data for this study were obtained from a larger study where the focus was not attention per se, there were no details available about diagnosis of or suspected ADHD, nor if any of the children were prescribed medication for ADHD. However, the participants in this study were on average aged 6 years and 6 months. Guidance in the United Kingdom suggests that diagnosis and medication in children under the age of 5 years only occurs in extreme cases, and the waiting lists experienced (especially around the time of this study which was impacted by the COVID pandemic) mean that it is unlikely many children in this study will have had a definitive diagnosis.^8,51^ While information on suspected diagnosis would have been interesting to add to this study, it was not the focus and did not detract from being able to describe the attention behaviors observed.

## Implications for Practice and Research

Speech and language pathologists working in the field of CLP will be aware of attention difficulties in some of the children they see whether they have a diagnosis of ADHD or not. This paper helps us to understand the nature of attention difficulties in this population and their potential link with speech and language development. Further research is needed to fully understand the relationship, and the impact early attention difficulties might have on speech and language development. Future research should focus on development, quantifying attention, and listening skills alongside speech, language, and hearing longitudinally.

## Conclusion

Standard scores on an assessment of attention in this group of children with CLP were low for the ability to focus on both auditory and visual stimuli. This raises the question of potential generalized attention difficulties in children with CLP. Both auditory and visual attention skills were correlated with language skills and this relationship needs to be explored further. Clinically, speech and language pathologists and other professionals working with children with CLP should be aware that attention difficulties are prevalent and advise families and educators accordingly. Future intervention studies would allow us to investigate the impact of advice and treatment on both attention and language development.
